# A boundary enhanced multi-task neural attention approach for Chinese named entity recognition

**DOI:** 10.1038/s41598-025-25317-5

**Published:** 2025-11-21

**Authors:** Jun Pan, Mingcheng Xiao, Mengpei Li, Feiyu Hu

**Affiliations:** 1 School of Science, Zhejiang University of Science and Technology, Hangzhou, 310023 China; 2 School of Sustainability and Tourism, Ritsumeikan Asia Pacific University, Beppu, 8748577 Japan

**Keywords:** Multi-task learning, Convolutional attention network, Encoder-decoder, Chinese named entity recognition, Computer science, Information technology, Statistics

## Abstract

Named Entity Recognition (NER) stands as a fundamental task in Chinese information processing. However, it encounters unique difficulties due to the lack of explicit word boundaries in the Chinese language. This study proposes framing Chinese NER as a joint task that combines boundary detection and entity identification within an encoder-decoder architecture. The presented method utilizes hybrid embeddings to enhance word-level representations and naturally incorporates head and tail boundary information to improve NER performance. It combines two types of tasks: sequence labeling for NER and binary classification for boundary prediction. In the primary NER task, a convolutional attention network serves as the encoder to extract contextual information about the target word from the input. For the auxiliary boundary prediction task, two Bi-GRU networks are employed to model long range semantic associations and predict the start and end of entities. A feature fusion layer is then introduced to adjust the contribution of the main and auxiliary tasks to the hidden states of the global representation. The final input representation, obtained through the joint training framework where the learned boundary information supports the NER task, is passed to the CRF decoding layer. Experimental results on the Weibo and Ontonotes5.0 datasets show that the multi - task learning framework significantly enhances Chinese NER performance compared to existing models.

## Introduction

Named Entity Recognition (NER) aims to extract specific meaningful entities from text and categorize them into predefined classes. As a foundational upstream task in natural language processing (NLP), NER plays a critical role in downstream applications such as entity linking^1^, machine translation^2^, relation extraction^3^, etc. Compared to English, Chinese lacks explicit word boundaries, which complicates the determination of entity boundaries and represents a significant challenge for Chinese NER systems. Multi-task learning (MTL) involves the simultaneous optimization of multiple objective functions, in which shared knowledge across tasks can mutually enhance performance. Given that identifying the start and end position information of entities is fundamental to entity recognition, we employ a multitask framework to leverage boundary information generated from entity boundary prediction, thereby improving NER performance through complementary task interactions.

Although some studies^4–6^ primarily focus on training deep language models based on character embedding, prior research has demonstrated that lexical information rich in prior linguistic knowledge holds significant potential to enhance NER performance. Word-level sequences can provide richer boundary cues for named entity recognition^7^. Motivated by this, we integrate character-level and word-level information to strengthen the model’s text representation capability.

**Fig. 1:**
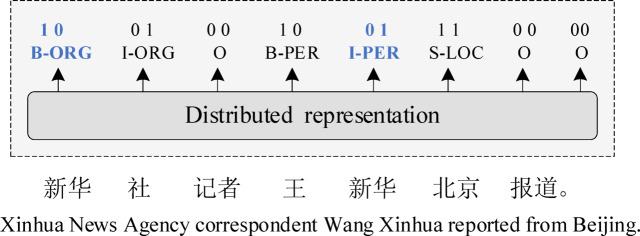
Example of Chinese entities with BIO labeling, where “ORG”, “PER”, and “LOC” represent pre-defined entity categories, while the label “10” indicates that the token is an entity head but not a tail.

A central challenge in Chinese NER is entity boundary ambiguity, where the semantic category of a token depends on its positional role (start or end) within an entity. For instance, “新华 (Xinhua)” represents an organization when it is the start of an entity (“新华社 Xinhua News Agency”) but a person when it is the end of an entity (“王新华 Wang Xinhua”) (see Fig. 1). Traditional methods treat NER as a pure sequence labeling task and fail to explicitly encode boundary position information, leading to ambiguous judgments of entity types.

To effectively exploit both entity boundary information and word-character information for NER enhancement, we propose a multi-task learning framework that fuses word-character representations and leverages boundary information derived from entity boundary prediction as shared knowledge. For the primary NER task, we employ a Convolutional Attention Network (CAN) encoder to capture contextual information of central words. For auxiliary tasks, we formulate entity start index prediction and entity end index prediction as two binary classification subtasks. A feature fusion layer is designed to dynamically adjust the relative importance of task-specific information in the hidden features. Finally, a Conditional Random Field (CRF) decoder is adopted to predict entity labels.

In summary, we make the following contributions in this paper:

1. We propose a novel and effective learning approach that frames Chinese NER as a joint task of boundary detection and entity identification within an encoder-decoder architecture.
2. A convolutional attention network is introduced to capture local contextual semantics, while Bi-GRU networks model long-range boundary dependencies, enabling dynamic feature fusion for improved representation.
3. Extensive experiments demonstrate that BEM-NER outperforms state-of-the-art models by explicitly leveraging entity boundary information, particularly in resolving ambiguous entity types.

The rest of this paper is structured as follows: Sect. 2 reviews the related studies on Chinese NER. Section 3 details the proposed methodology. Section 4 presents the experimental results and evaluates the performance on both the Weibo dataset and the OntoNotes 5.0 dataset. Finally, Sect. 5 summarizes the research findings and outlines the future research directions.

## Related work

Named entity recognition is commonly approached as a sequence labeling task, where each token in a text sequence is assigned a semantic label denoting its entity category or non-entity status. Early approaches relied on rule-based systems, which leverage linguistic expertise to design handcrafted patterns for entity matchings^8^. However, these approaches suffered from limited scalability and required extensive manual annotation effort, prompting their gradual replacement by statistical machine learning techniques. The latter learn discriminative patterns from labeled data to predict entity boundaries and types^9^, marking a pivotal shift toward data-driven NER.

The advent of deep learning has revolutionized NER by enabling automatic extraction of hierarchical semantic features. Neural architectures such as Convolutional Neural Networks (CNNs), Recurrent Neural Networks (RNNs), Graph Neural Networks (GNNs), and Transformers have been widely adopted to model contextual dependencies^10^. Unlike traditional machine learning methods that rely heavily on manually engineered features, deep learning frameworks excel at capturing multi-level linguistic structures, driving significant advancements in NER performance.

Given that both lexical and character-level information contribute to NER performance enhancement, numerous hybrid approaches integrating character and word representations have been developed in recent years^10–16^. Notably, Zhang et al.^12^ advanced the Bi-LSTM architecture by incorporating lattice neural units with lexical information, enabling the integration of word-level cues into hidden states through character sequence modeling. In a similar vein, Liu et al.^13^ proposed the WC-LSTM model, which injects word information into the start or end characters of words to explicitly capture word boundary cues. To simplify lexicon integration, Ma et al.^14^ introduced a method that categorizes matched words into four groups based on character positions within the word, using a weighting mechanism to compute word set representations. Addressing structural challenges in lattice-based models, Li et al.^15^ developed a flattening strategy that converts lattice structures into linear sequences by assigning dual positional indices to each word element. This approach effectively combines word information capture with positional encoding, utilizing spacing between indices to represent lexical context. Gui et al.^16^ further innovated with the LR-CNN model, which combines lexicon and adds a feedback layer to the CNN network that uses high-level features to adjust the weights of character representation.

Multi-task learning enhances model generalization by leveraging shared information across tasks, making it a promising paradigm for entity recognition^17–21^. In recent years, numerous studies have explored MTL-based named entity recognition models, aiming to improve NER performance by integrating auxiliary tasks. Notably, the Chinese word segmentation (CWS) task which provides explicit word boundary information has been widely adopted as an auxiliary task. Peng et al.^7^ employed an LSTM-CRF architecture to jointly train NER and CWS tasks, sharing word boundary information across tasks. Their approach yielded significant performance gains in NER, particularly on Chinese social media texts, highlighting the utility of cross-task boundary cues. Building on this, Cao et al.^20^ proposed an MTL framework that incorporates CWS and introduced adversarial training to effectively extract NER-specific shared features from the CWS task, further enhancing the model’s ability to exploit task correlations. Beyond CWS, part-of-speech (POS) tagging, which provides valuable syntactic information, has been shown to benefit NER. Leveraging this insight, Zhu et al.^21^ developed a multi-task framework that integrates CWS with POS tagging. In their model, the POS task acts as an auxiliary component, contributing additional linguistic features (e.g., syntactic roles) to strengthen NER performance through multi-level information fusion. These works collectively demonstrate the value of MTL in harnessing complementary task information to address the challenges of Chinese NER. However, these models still faced challenges from shared parameter conflicts and residual segmentation errors affecting NER accuracy.

A growing number of works emphasize the critical role of entity boundary detection in resolving Chinese NER ambiguities. Zheng et al.^22^ proposed a boundary-aware model using region-based features to predict entity types, reducing hierarchical labeling errors. Chen et al.^23^ introduced graph attention networks to model intra-entity semantic dependencies, treating boundary prediction as an auxiliary task. Liu et al.^24^ designed a label-wise Transformer that separates boundary and type prediction across network layers, improving modularity. Despite these advances, existing methods often underutilize the dual semantic role of boundary tokens, leading to ambiguous representations during boundary identification.

In summary, current approaches either focus on lexical integration without explicit boundary modeling or treat boundary information as implicit cues. However, in Chinese texts, character-level boundary ambiguity remains a persistent challenge, as entity types are highly dependent on positional roles. To address this, we propose modeling boundary detection as an explicit auxiliary task, enabling the model to learn discriminative boundary features while dynamically fusing character and word level information through adaptive feature weighting.

## The BEM-NER model

Entity boundary information refers to the positional information of entity phrases within the text sequence, i.e., the start-to-end positional range of entity phrases in the text sequence. We explicitly model entity boundary detection as an auxiliary task alongside the primary NER task. This dual-task architecture leverages multi-task learning to jointly optimize boundary localization and entity classification, enabling the model to capture positional semantics crucial for resolving structural ambiguities in Chinese text.

### System overview

**Fig. 2:**
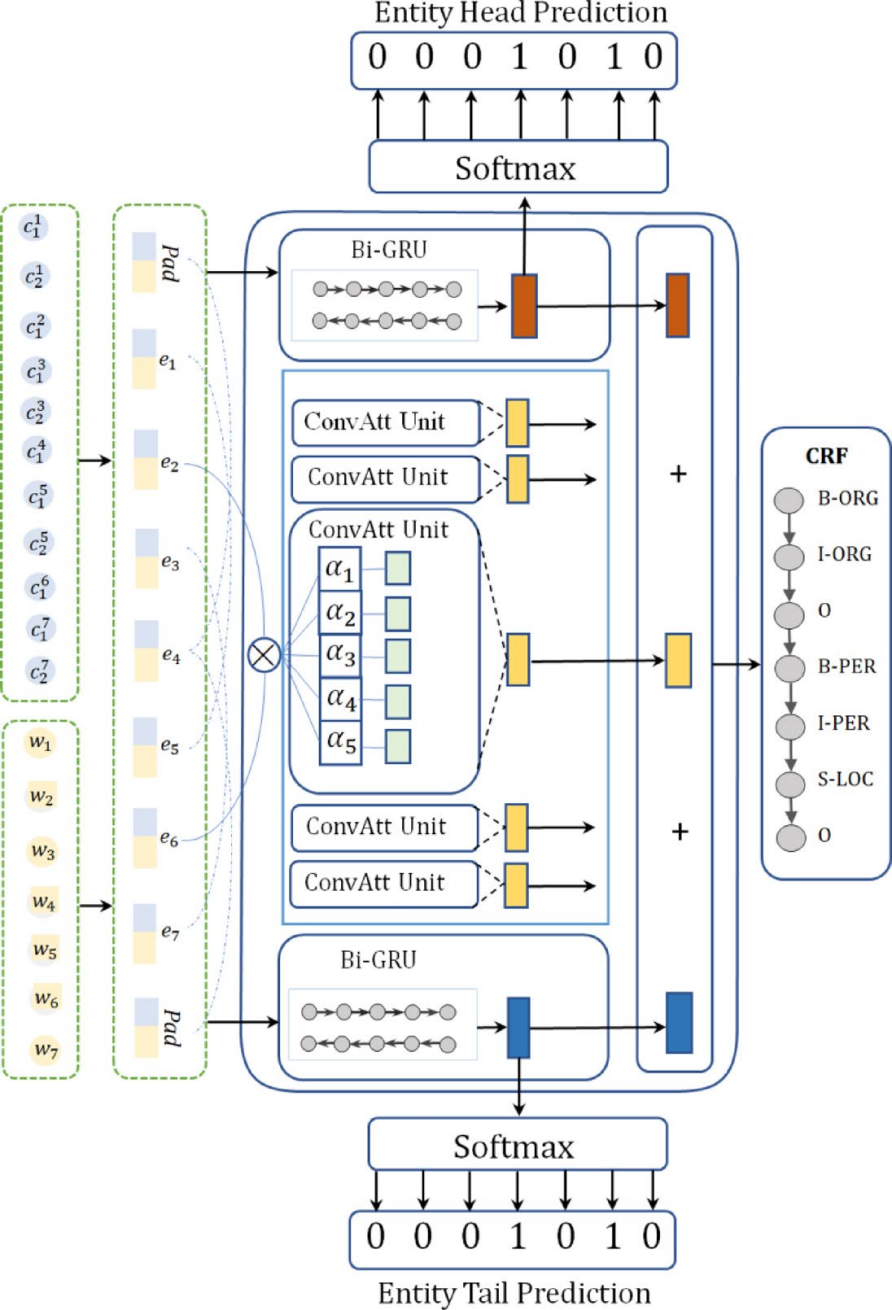
The overall framework of the BEM-NER model.

The architecture of our proposed model is illustrated in Fig. 2. It comprises two core components: a primary NER task module and an auxiliary entity boundary prediction subtask module. The overall framework can be decomposed into five sequential layers: a word-character embedding layer, a Bi-GRU-based entity start/end index encoding layer, a convolutional attention network (CAN) context encoding layer, a feature fusion layer, and a decoding layer.

First, we fuse the lexical information from the original text with corresponding character-level representations to generate multi-grained input embeddings, capturing both fine-grained character semantics and coarse-grained word context. Subsequently, the Bi-GRU network processes the embedded representations to encode positional features of entity start and end indices. This layer focuses on capturing sequential dependencies critical for boundary localization, generating boundary-aware hidden states. Concurrently, the convolutional attention network (CAN) is employed to encode global contextual semantics. By leveraging convolutional kernels with attention mechanisms, CAN dynamically emphasizes task-relevant contextual features, complementing the sequential encoding from the Bi-GRU layer. The outputs from the Bi-GRU boundary encoding layer (containing explicit boundary position information) and the CAN context encoding layer (containing rich semantic context) are then fed into the feature fusion layer. This layer adaptively integrates multi-source features by adjusting the relative importance of boundary cues and semantic context through learnable weighting parameters. Finally, The Bi-GRU boundary encoding output is decoded using a softmax classifier to predict entity start/end indices, and the fused features from the fusion layer are decoded via a conditional random field to generate final entity labels.

### Embedding representation layer

Before the sequence can be contextually encoded, it must first be converted into distributed representations that properly reflect the semantic and syntactic characteristics of the input text. In this study, we approach NER as a word-level sequence tagging task, where each word in the input sequence is initially embedded in an N-dimensional space.

However, in the Chinese language, characters serve as the fundamental units, each carrying its own meaning and possessing the ability to form words independently. Hence, it is crucial to consider the constituent characters of a word, as many Chinese entities are compound words whose meanings are derived from their constituent characters. Moreover, entities of the same category may share common characters, highlighting the importance of exploiting the rich semantics embedded in characters. Furthermore, for infrequently used words with limited training data, their meanings can be enriched by incorporating the embeddings of their constituent characters. Therefore, we enrich the word representations with character-level information and utilize this information within the word through a convolutional network to extract local features around each character.

To exploit both word-level and character-level information, we employ commonly used pre-trained Chinese embeddings sourced from the large unlabeled text via literal matching^25^. The character-level embeddings of each word are initially passed on to a CNN network to capture intra-word features. The final global feature vector is generated by combining the word-level and character-level embeddings.

For a given sequence of raw input words $\:S=\left\{{w}_{1},{w}_{2},\cdots\:,{w}_{n}\right\}$, where $\:{w}_{i}$ represents the $\:i$-th word$\:,$ we first obtain the word representation using pre-trained vectors, denoted as $\:{x}_{i}^{w}={\text{e}}^{\text{w}}\left({w}_{i}\right)$, where$\:\:{\text{e}}^{\text{w}}$ represents a word embedding lookup table. Assuming $\:{w}_{i}=\left\{{c}_{1}^{i},{c}_{2}^{i},\cdots\:,{c}_{m}^{i}\right\}$, where $\:{c}_{j}^{i}$ signifies the $\:j$-th character of $\:{w}_{i}$, each $\:{c}_{j}^{i}\:$is encoded as $\:{x}_{j}^{c}={\text{e}}^{\text{c}}\left({c}_{j}\right)$, where $\:{\text{e}}^{\text{c}}$ denotes a character embedding lookup table. We then fed the character sequence embedding of the word $\:{w}_{i}$, i.e., $\:\left\{{x}_{1}^{c},{x}_{2}^{c},\dots\:,{x}_{m}^{c}\right\}$, into a CNN network to produce an intra-word feature representation:

1 $$\:{h}_{i}^{c}=\text{C}\text{N}\text{N}\left({x}_{i}^{c}\right),$$

**Fig. 3:**
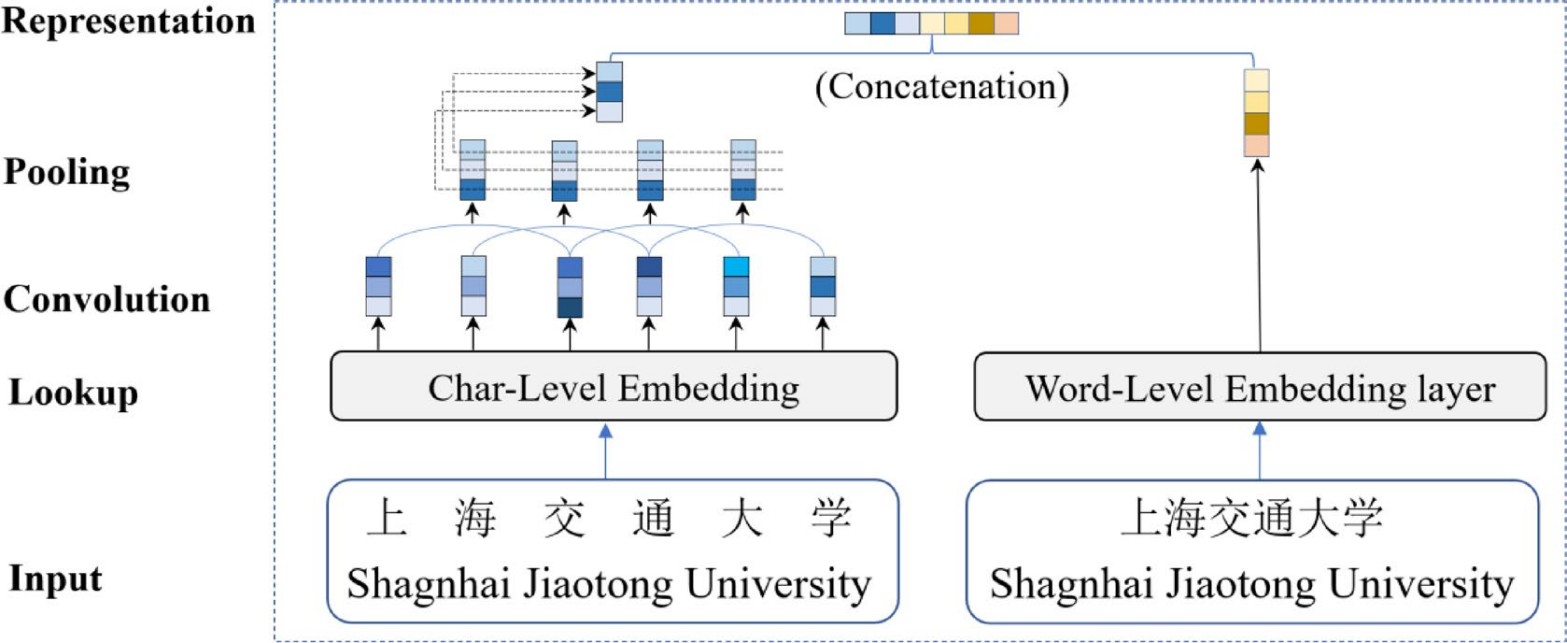
An example of hybrid representation.

The final input is obtained by concatenating the word embeddings with the character representation of the word extracted by the CNN:

2 $$\:{x}_{i}=\left[{x}_{i}^{w}\oplus\:{h}_{i}^{c}\right],$$

where ⊕ represents the vector concatenation operation, as shown in Fig. 3.

### Context encoder

The context encoder is designed to model sequential dependencies in input data and produce context-aware representations for downstream label prediction tasks. While recurrent neural networks (RNNs), convolutional neural networks (CNNs), and graph neural networks (GNNs) have been traditionally employed for this purpose, recent work has demonstrated the advantages of transformer architectures in capturing global dependencies through self-attention mechanisms. Inspired by the local attention framework proposed in^4^, we introduce a convolutional attention layer to model implicit relationships between adjacent tokens. By fusing word segmentation vectors with character embeddings, this layer semantically groups related tokens. Additionally, two BiGRU modules are employed to capture sentence-level dependencies, enhancing entity boundary detection.

#### Convolutional attention networks

Effective context encoding requires a balance between local semantic granularity and global dependency modeling. Traditional sequence models like RNNs suffer from gradient vanishing in long sequences and lack parallel processing capabilities. Pure CNNs excel at parallel computation but struggle with non-local interactions, while transformers excel at global attention but incur high computational costs, especially on small datasets. We introduce a convolutional attention layer that integrates local attention mechanisms into CNNs, enabling efficient capture of implicit contextual relationships between adjacent tokens.

The CNNs generates a fixed-size vector representation of the structure by consolidating these patterns, capturing the most relevant local features for the specific prediction task. The convolutional attention layer encodes the input sequence, grouping related tokens within the local context implicitly.

We set the window size of the CNN to $\:j$. The window centered on the $\:k$-th token of the input sequence is represented as $\:{\varvec{x}}_{k}=\{{x}_{k-\frac{j-1}{2}},\cdots\:,{x}_{k},\cdots\:,{x}_{k+\frac{j-1}{2}}\}\:$, where $\:{x}_{k}$ denotes the concatenated character and word embedding, with a dimension denoted as $\:{d}_{input}$. Within this window, we then apply local attention to capture the relations between the center token and its context. The hidden dimension of this layer is denoted as $\:{d}_{h}$. For the $\:k$-th token, the local attention takes $\:{W}_{k}$ as its input, and outputs $\:j$ hidden vectors as follows:

3 $$\:{g}_{m}={\alpha\:}_{m}{x}_{m},$$

where $\:m\in\:E,E=\left\{k-\frac{j-1}{2},\dots\:,k+\frac{j-1}{2}\right\}$, and $\:{\alpha\:}_{m}$ is the attention weight, computed by the following formula:

4 $$\:{\alpha\:}_{m}=\frac{\text{e}\text{x}\text{p}\:s\left({{x}_{k},\:\:\:x}_{m}\right)}{{\sum\:}_{i\in\:E}\text{e}\text{x}\text{p}\:s\left({{x}_{k},\:\:\:x}_{i}\right)},$$

where the attention score function $\:\text{s}$ measures the correlation between $\:{x}_{k}$ and $\:{x}_{m}$. A higher value indicates that elements close to position $\:m$ carry more information and should be weighted more. The formula for computing $\:\text{s}$ is as follows:

5 $$\:\text{s}\left({x}_{k},{x}_{m}\right)={V}^{\text{T}}\text{tanh}({W}_{1}{x}_{k}+{W}_{2}{x}_{m}),$$

where$\:\:{W}_{1}$,$\:{W}_{2}$,$\:V$ are trainable parameter vectors of the model and $\:V\in\:{\text{R}}^{{d}_{h}}$, $\:{W}_{1}$,$\:{W}_{2}\in\:{\text{R}}^{{d}_{h},{d}_{input}}$. The CNN layer employs $\:{d}_{h}$ filters in a contextual window of size $\:j$. It uses a range of filters with various window sizes for learning contextual information. When the input$\:\:{G}_{k}=\left\{{g}_{k-\frac{j-1}{2}},\cdots\:,{g}_{k},\cdots\:,{g}_{k+\frac{j-1}{2}}\right\}$ is fed into the CNN with the sum pooling layer, we have

6 $$\:{h}_{k}^{acn}={\sum\:}_{j}\left({W}^{acn}\text{*}{h}_{k-\frac{j-1}{2}:k+\frac{j-1}{2}}\right)+{b}^{acn},$$

where $\:{W}^{acn}\in\:{\text{R}}^{{j\times\:d}_{h}\times\:{d}_{input}}$, $\:{b}^{acn}\in\:{\text{R}}^{{j\times\:d}_{h}}$.The ∗ operation denotes element-wise product, and $\:{h}_{k-\frac{j-1}{2}:k+\frac{j-1}{2}}$ denotes the concatenation of the hidden states $\:{h}_{i\in\:E}$. The elements in the same dimension are multiplied first, and in order to sum up the variables within each pooling window, a summation pooling operation is then conducted in this dimension. Finally, we obtain the output of the convolutional attention layer, i.e., $\:{H}_{ACN}=\left\{{h}_{1}^{acn},{h}_{2}^{acn}\cdots\:,{h}_{n}^{acn}\right\}$, and $\:{h}_{i}\in\:{\text{R}}^{{d}_{h}}$.

#### BI-GRU-based entity boundary encoding

Although the convolutional attention network successfully captures semantic relations between neighboring words, there is still a need to strengthen the boundary of the entity. In this study, to provide distinct entity boundary information for NER, we treat entity boundary detection as a binary classification task and train it simultaneously with NER.

The GRU network is a specialized type of RNN designed to address issues related to long-range dependencies and gradient explosions often faced by traditional RNNs. It has demonstrated superior performance in various NLP tasks. In contrast to LSTM, which utilizes multiple gating mechanisms, GRU accomplishes memory forgetting and filtering using a single gating mechanism. This streamlined approach simplifies the network architecture, reduces parameter count, and enhances efficiency. Therefore, we utilize two distinct layers of GRU networks to encode the hybrid input representation, effectively capturing long-term dependencies within the text sequence to facilitate subsequent computations.

For each time frame, the GRU network makes efficient use of information from both the past (via forward states) and the future (via backward states). It generates a final hidden representation by concatenating the hidden states obtained from opposite directions. Given sentence representations $\:\varvec{x}=\left\{{x}_{1},{x}_{2},\cdots\:,{x}_{n}\right\}$, the hidden state of token $\:{x}_{i}\:$encoded by the bidirectional GRU network is computed as follows:

7 $$\:{\overrightarrow{h}}_{i}=\overrightarrow{\text{G}\text{R}\text{U}}\left({x}_{i},{\overrightarrow{h}}_{i-1}\right),$$

8 $$\:{\overleftarrow{h}}_{i}=\overleftarrow{\text{G}\text{R}\text{U}}\left({x}_{i},{\overleftarrow{h}}_{i-1}\right),$$

9 $$\:{h}_{i}=\left[{\overrightarrow{h}}_{i}\oplus\:{\overleftarrow{h}}_{i}\right],$$

where the symbol ⊕ denotes concatenation, containing evidence from the entire input sentence. Subsequently, the two bidirectional GRU networks generate representations for head prediction ($\:{H}_{head}$) and tail prediction ($\:{H}_{tail}$) respectively. These encoded representations are then inputted into the classifiers of a multilayer perceptron (MLP) and softmax layers, which detect whether the word is the beginning or the end of an entity, ensuring that the hidden state $\:{h}_{i}$ carries head and tail information.

### Global feature fusion

The feature fusion layer plays a crucial role in integrating data from both the primary and auxiliary tasks through the utilization of learnable parameters. It combines the local feature vector ($\:{H}_{ACN}$) extracted by the convolutional attention network, with the features learned from the two Bi-GRU networks in the auxiliary tasks ($\:{H}_{head}$ and $\:{H}_{tail}$) to form the global feature vector.

During the training process, the hidden layers produce predictions for the three tasks with the aim of aligning with the actual labels. The feature fusion layer can be computed by the following equation:

10 $$\:H={W}_{4}\cdot\:{H}_{ACN}+{W}_{5}\cdot\:{H}_{head}+{W}_{6}\cdot\:{H}_{tail},$$

where $\:{W}_{4}$, $\:{W}_{5}$, and $\:{W}_{6}$ are the trainable parameter vectors of the model. Finally, these global features of fixed size are fed into a standard CRF decoder to calculate distribution values for all potential tags of the input data. This mechanism allows the model to learn task-specific and shared representations simultaneously, improving generalization for both NER and boundary detection.

### Tag decoder

#### Boundary detection

We employ a BI-GRU model that receives the encoded words as input and generates a hidden state for each sentence. These hidden states encapsulate crucial contextual information about the words. Following this, the hidden states of the encoded sentences are inputted into an MLP classifier for further processing.

More specifically, the encoded contextual representation $\:{h}_{i}$ is used to compute the probability of the word $\:{w}_{i}$ appearing in either the head or tail position of an entity, respectively:

11 $$\:{P}_{head}^{i}=\text{s}\text{o}\text{f}\text{t}\text{m}\text{a}\text{x}\left({\text{M}\text{L}\text{P}}_{\text{h}\text{e}\text{a}\text{d}}\left({h}_{i}\right)\right)$$

12 $$\:{P}_{tail}^{i}=\text{s}\text{o}\text{f}\text{t}\text{m}\text{a}\text{x}\left({\text{M}\text{L}\text{P}}_{\text{t}\text{a}\text{i}\text{l}}\left({h}_{i}\right)\right)$$

#### Named entity recognition

The label sequence is a vital aspect of NER due to the strong dependencies among entity labels. For example, ‘B-LOC’ may be succeeded by ‘I-LOC’ but not ‘I-ORG’, and must be the first label of an entity. To capture these kinds of dependencies, we employ conditional random fields, a widely used decoder for structured output.

The CRF is a global random field that is conditioned on the sequence of observations. By taking into account neighboring tagging information, it enables the extraction of additional label transfer details to enhance sequence labeling. The CRF is utilized on top of the feature fusion layer to facilitate the primary task of Named Entity Recognition (NER), calculating the probability of the sequence of the true labels.

Given a raw sentence $\:S$ with words $\:{\left\{{w}_{i}\right\}}_{i=1}^{l}$, the input to the CRF layer is represented as $\:\varvec{x}=\:{\left\{{x}_{i}\right\}}_{i=1}^{l}$, while the predicted sequence of labels for the given sentence is denoted as $\:\mathbf{y}={\left\{{y}_{i}\right\}}_{i=1}^{l}$. The corresponding probabilities are calculated as follows:

13 $$\:f\left(\varvec{x},\varvec{y}\right)=\sum\:_{i=1}^{l}{T}_{{y}_{i},{y}_{i+1}}+\sum\:_{i=1}^{l}{P}_{i,{y}_{i}},$$

14 $$\:P\left(\varvec{y}\right|\varvec{x})=\frac{\text{e}\text{x}\text{p}\left(f\left(\varvec{x},\varvec{y}\right)\right)}{{\sum\:}_{{\varvec{y}}^{\mathbf{{\prime\:}}}}\text{e}\text{x}\text{p}\left(f\left(\varvec{x},{\varvec{y}}^{\mathbf{{\prime\:}}}\right)\right)},$$

where $\:f\left(\varvec{x},\varvec{y}\right)\:$computes the score of path $\:\varvec{y}$ on input sequence $\:\varvec{x}$, $\:{T}_{{y}_{i},{y}_{i+1}}\:$denotes the score of a transition from $\:{y}_{i}$ to $\:{y}_{i+1}$, and $\:{P}_{i,{y}_{i}}$ is the score of the $\:{y}_{i}\:$tag of the $\:i$-th word, $\:{\varvec{y}}^{\mathbf{{\prime\:}}}$ denotes all possible candidate output. The search space of the candidates is so large that an exact inference is very difficult, even by means of dynamic programming. Hence, we use the first-order Viterbi algorithm^26^ to search for the highest-scoring output during training and testing.

### Multi-task training

The learning task in this study involves mapping a candidate output y to a global feature vector. Parameter values are set during the training process using the training examples provided. A vital aspect of this process is the gradient reversal layer, responsible for updating the shared information extractor through gradient reversal. The goal is to accurately identify the boundary of the entity and the tags of an input sentence. Once trained, contextualized word embeddings containing common information are extracted from hidden states. These embeddings are subsequently utilized for multi-task learning, enhancing the model’s performance across all three tasks.

We use three loss functions to optimize the model. Formally, denote a training set as $\:D={\{{\varvec{x}}_{n},{(\varvec{y}}_{n}^{ner},{\varvec{y}}_{n}^{s},{\varvec{y}}_{n}^{e})\}}_{n=1}^{N}$, where each training instance consists of an input sequence $\:\varvec{x}$ and its corresponding NER labels $\:{\varvec{y}}^{ner}$, entity head labels $\:{\varvec{y}}^{s}$ and tail labels $\:{\varvec{y}}^{e}$. For the primary task, we use the loss function of the log-likelihood function to optimize the model:

15 $$\:{\text{L}}_{\text{m}\text{a}\text{i}\text{n}}=-\frac{1}{\left|D\right|}\sum\:_{n=1}^{N}\text{l}\text{o}\text{g}\text{p}\left({(\varvec{y}}_{n}^{ner}\right|{\varvec{x}}_{n}).$$

By minimizing this loss function, the model will be aligned with the actual labels. The backpropagation algorithm calculates the gradients of the loss function about the model parameters, enabling iterative learning and improved performance. By minimizing the loss function of the negative log-likelihood, BEM-NER optimizes its parameters and generates precise sequences of labels for named entity recognition.

For the entity boundary prediction task, we use the following two cross-entropy losses to detect the head and tail boundaries respectively,

16 $$\:{\text{L}}_{\text{s}}=-\frac{1}{\left|D\right|}\sum\:_{n=1}^{N}\sum\:_{i=1}^{l}{(\varvec{y}}_{n,i}^{h}\text{log}{P}_{s}^{n,i}+(1-{\varvec{y}}_{n,i}^{e})\text{l}\text{o}\text{g}(1-{P}_{s}^{n,i})),$$

17 $$\:{\text{L}}_{\text{e}}=-\frac{1}{\left|D\right|}\sum\:_{n=1}^{N}\sum\:_{i=1}^{l}{(\varvec{y}}_{n,i}^{e}\text{log}{P}_{s}^{n,i}+(1-{\varvec{y}}_{n,i}^{e})\text{l}\text{o}\text{g}(1-{P}_{e}^{n,i})),$$

where $\:n,i$ denotes the $\:i$-th token of the $\:n$-th sentence. For training, we jointly minimize the following loss of BEM-NER:

18 $$\:\text{L}={\text{L}}_{\text{m}\text{a}\text{i}\text{n}}+{\text{L}}_{\text{s}}+{\text{L}}_{\text{e}}.$$

## Experiments

To evaluate the effectiveness of our joint entity boundary detection-based NER method, we conducted comprehensive experiments. We used the standard OntoNotes 5.0 and Weibo datasets and compared our approach against classical NER baseline models. Through ablation studies, we observed the benefits of incorporating shared boundary information or adopting character-word fusion strategies. Additionally, we investigated the impact of different annotation schemes on the model, and conducted comparative experiments on various boundary information fusion methods to explore further performance improvements.

The model parameters were optimized using the Adam optimizer. The training environment consisted of an NVIDIA 3090 with CUDA 11.6.1, and the code was implemented on the NCRF + + framework (https://github.com/jiesutd/NCRFpp). Using a controlled variable method, we evaluated the performance of 5 parameter combinations on the dataset. Results indicate that the selected parameter combination yields the highest average F1 score, with detailed hyperparameters listed in Table 1.

**Table 1:** The detail hyperparameters.

Hyperparameters	Values
Dropout rate	0.5
Learning rate on Weibo	0.0005
Learning rate on Ontonotes5.0	0.0005
Batch size	32
Convolution window	5
Word vector dimension	300
Character vector dimension	300
Learning rate decay	0.05
Hidden layer dimension	300

### Datasets

The Weibo dataset was obtained from the social media platform Sina Weibo, encompassing four main entity categories: individuals, places, groups, and geopolitics. For consistency, we adopted the data division approach used in^7^. The OntoNotes5.0 dataset, on the other hand, was gathered from Chinese news and broadcasting sources, featuring 18 distinct entity classifications such as individuals, groups, and places^27^. We use the same division approach as in^28^. Table 2 provides an overview of the division statistics of the two datasets. Notably, all datasets were annotated following the ‘BMESO’ tagging scheme, which designates the beginning (B), middle(M), end (E), single (S), and outside (O) tags for entity recognition.

**Table 2:** Dataset division statistics (unit: sentence).

Dataset	Train	Dev	Test
Ontonotes5.0	36,487	6,083	4,472
Weibo	1350	270	270

### Evaluation metrics

NER performance was evaluated using standard metrics, namely precision (P), recall (R), and F1 score (F1). Precision is calculated as the ratio of correctly predicted entities to all recognized entities, while recall is the ratio of correctly predicted entities to all target entities. The F1 score, being the harmonic mean of recall and precision, provides a balanced measure of performance. The formulas for the P, R, and F1 scores are as follows:

19 $$\:\text{P}=\frac{\text{T}\text{P}}{\text{T}\text{P}+\text{F}\text{P}}\times\:100\%,$$

20 $$\:\text{R}=\frac{\text{T}\text{P}}{\text{T}\text{P}+\text{F}\text{N}}\times\:100\text{\%},$$

21 $$\:{\text{F}}_{1}=\frac{2\text{P}\text{*}\text{R}}{\text{P}+\text{R}}\times\:100\text{\%},$$

where TP refers to the number of positive examples correctly predicted, FP represents the number of examples incorrectly predicted as positive, and FN denotes the number of examples incorrectly predicted as negative.

### Results and analysis

#### Baselines

We conducted a comparative analysis of our proposed model against several existing baselines, including some recent advanced Chinese NER models, to evaluate its efficacy.

1. Zhang et al.^12^: They propose the Lattice-LSTM model, which encodes a sequence of input characters and potential words from a word lexicon. This integration of word information helps avoid word segmentation errors.
2. Cao et al.^20^: They presented a multi-task learning framework that incorporates Chinese word segmentation (CWS) tasks. Through adversarial training, they successfully acquired task-shared boundary information, leading to enhanced Chinese NER performance.
3. Liu et al.^13^: They propose the WC-LSTM model, which incorporates word information into either the head or the tail of a word to capture boundary information effectively.
4. Ma et al.^14^: They propose a simple model that incorporates lexicon information. This approach determines the character’s position within a matching word, categorizes matching words into four BMES sets, and computes lexicon representation using a weighting system.
5. Li et al.^15^: They propose the FLAT model, which utilizes a flat-lattice Transformer to integrate lexicon information into Chinese NER. By transforming the lattice structure into spans and employing a specific positional encoding strategy, this model maximizes the utilization of lattice information and exhibits strong parallelization capabilities.
6. Zhu et al.^21^: They proposed a multi-task learning framework that utilizes Chinese word segmentation and POS tagging tasks as auxiliary tasks to provide additional syntactic information for named entity recognition.
7. Jie et al.^29^: They incorporated additional syntactic dependency tree information into word combinations to enhance entity recognition performance.

#### Main results

*Weibo*: Table 3 presents the performance of our BEM-NER model on the Weibo dataset. The dataset contains four entity categories, categorized into named entities (NE) and nominal mentions (NM). Specifically, named entities refer to unique entities in the dataset, such as Weibo usernames and specific organizations, while nominal entities refer to general entities like “singer” and “palace”, among others. The results indicate that Our model achieves the highest overall F1 score (64.47%), outperforming existing methods. Notably, it improves the F1 metric by 5.68% compared to the traditional lattice-LSTM model.

**Table 3:** Performance on Weibo dataset.

Models	NE	NM	Overall
	F1 (%)	F1 (%)	F1 (%)
Zhang et al.^12^	53.04	62.25	58.79
Cao et al.^20^	54.34	57.35	58.70
Liu et al.^13^	52.55	**67.41**	59.84
Ma et al.^14^	56.99	61.41	61.24
Li et al.^15^	-	-	63.42
Zhu et al.^21^	**62.94**	64.40	63.65
BEM-NER	61.30	66.67	**64.47**

*OntoNotes5.0*: Table 4 summarizes the results on the OntoNotes5.0 dataset. The lattice-LSTM model achieves an F1 score of 76.67%, while the DGLSTM-CRF model (which incorporates syntactic dependency tree information) reaches 77.40%. Our BEM-NER model further improves performance, achieving 77.78% F1, a 1.11% increase over lattice-LSTM and 0.38% over DGLSTM-CRF.

**Table 4:** Performance on OntoNotes5.0 dataset.

Models	OntoNotes5.0		
	*P* (%)	*R* (%)	F1 (%)
Zhang et al.^12^	76.34	77.01	76.67
Jie et al.^29^	77.40	77.41	77.40
BEM-NER	**77.93**	**77.64**	**77.78**

### Ablation study

To evaluate the contribution of individual components in BEM-NER, we conducted ablation experiments on two datasets by systematically disabling specific modules: (1) Baseline Model (CAN-only): A single-task model using only the Convolutional Attention Network (CAN) encoder without word-character fusion or boundary prediction auxiliary tasks. This serves as the foundational architecture for comparison. (2) MTL without Word-Character Fusion: A multi-task learning model incorporating entity boundary prediction but relying solely on character embeddings. This variant assesses the impact of word-character fusion on model performance. (3) Single-Task with Word-Character Fusion: A single-task model utilizing word-character fusion but excluding the entity boundary prediction auxiliary task. This evaluates the isolated contribution of boundary information to NER accuracy. (4) Full Proposed Model (MTL + Fusion):

The complete multi-task learning framework integrating both word-character fusion and entity boundary prediction. Table 5 presents the comparative results of these ablation experiments.

**Table 5:** Performance of ablation experiments.

Models	Weibo			OntoNotes5.0		
	*P* (%)	*R* (%)	F1 (%)	*P* (%)	*R* (%)	F1 (%)
BEM-NER - char & boundary info	71.38	51.11	59.57	76.92	71.80	74.27
BEM-NER - char info	69.84	54.32	61.11	76.09	75.31	75.70
BEM-NER - boundary info	71.29	55.80	62.60	76.34	77.37	76.93
BEM-NER -CAN only	66.32	62.72	64.47	77.93	77.64	77.78

Ablation study results demonstrate the critical contributions of both word-character fusion and entity boundary prediction. Comparative analyses between models with and without word-character fusion reveal that integrating lexical and character-level representations significantly enhances NER performance, with consistent improvements across both datasets. Specifically, the inclusion of shared boundary information or the adoption of word-character fusion leads to simultaneous increases in performance across both the Weibo and OntoNotes5.0 datasets. Notably, word-character fusion yields a 3.03% improvement in overall F1-score on the Weibo dataset and a 2.66% improvement on OntoNotes5.0 compared to models lacking this component, which validate its role in enhancing the overall model performance.

Additionally, the inclusion of boundary prediction in the multi-task framework yields statistically significant gains. The full model achieves 1.87% and 0.85% F1-score improvements on the Weibo and OntoNotes5.0 datasets, respectively, compared to variants lacking boundary information. Conversely, removing either component leads to significant F1-score degradation on both benchmarks, highlighting their indispensable roles in the architecture.

Notably, the Weibo dataset exhibits more pronounced improvements than OntoNotes5.0. This discrepancy can be attributed to the inherent characteristics of social media text in the Weibo corpus, which contains more out-of-vocabulary words, informal expressions, and ambiguous entity boundaries compared to the structured news domain of OntoNotes 5.0. In such low-resource, high-ambiguity scenarios, the shared boundary information from the auxiliary prediction task provides critical structural cues that mitigate uncertainty in entity localization. By contrast, the relatively regular syntax and explicit word boundaries in OntoNotes 5.0 reduce the dependency on external boundary cues, resulting in smaller but still significant performance gains.

Overall, these findings collectively validate the synergistic effects of multi-task learning with entity boundary prediction and word-character feature fusion. The complementary nature of lexical-semantic information from word-character fusion, and positional boundary cues from multi-task learning, demonstrates a robust solution to the challenges of Chinese NER, particularly in noisy, real-world datasets.

### Impact of different tagging schemes

The choice of tagging scheme in Chinese NER significantly influences model performance^30^. Two widely used schemes are BMESO and BIO. In BMESO, labels include B (Beginning of an entity), M (Middle of an entity), E (End of an entity), S (Single word entity) and O (Non-entity word). In contrast, the BIO scheme uses B, I (Inside or continuation of an entity) and O.

The granularity of a tagging scheme directly affects model efficiency and effectiveness. While fine-grained schemes like BMESO provide detailed boundary information, they increase computational complexity, particularly when training on large, fine-grained datasets. Conversely, coarser schemes like BIO reduce complexity but sacrifice boundary precision. To evaluate this trade-off, we transformed the Weibo and OntoNotes 5.0 datasets into three tagging formats: BMESO, BIO, and BIOS. The results are summarized in Table 6.

**Table 6:** Performance of different tagging schemes.

Tagging scheme	Weibo				OntoNotes5.0		
	*P*(%)	*R*(%)		F1(%)	*P*(%)	*R*(%)	F1(%)
BMESO	66.32	62.72		64.47	77.93	77.64	77.78
BIO	63.79	64.39		64.08	78.81	76.44	77.56
BIOS	66.49	62.22		64.29	78.19	77. 03	77.61

The results demonstrate that BMESO, the most granular scheme, achieves the highest F1 score on both datasets, confirming the value of explicit boundary modeling in Chinese NER. Specifically, BMESO outperforms BIO by 0.39% on Weibo and 0.22% on OntoNotes 5.0, while surpassing BIOS by 0.18% on Weibo. This highlights the benefits of capturing precise entity boundaries, particularly in Chinese NER, where morphological ambiguity and nested structures are common. However, the marginal gains of BIOS over BIO suggest that incorporating single-word entity labels (S) can still improve performance without the full complexity of BMESO.

### Comparison of different boundary information fusion methods

To systematically analyze the impact of boundary information fusion strategies, we conducted comparative experiments on four distinct fusion methods: (1) Fixed-Weight Start Position Fusion: This method combines features encoding entity start position information using predefined fixed weights, without parameter learning during training. (2) Fixed-Weight End Position Fusion: Similar to the first method, this approach fuses features containing entity end position information using manually specified fixed weights, lacking adaptive weight adjustment. (3) Fixed-Weight Joint Position Fusion: Here, we integrate both entity start and end position features using fixed weights, treating start/end boundary information as equally important a priori. (4) Learned-Weight Adaptive Fusion: In this approach, the fusion weights are model parameters optimized during training, allowing the framework to automatically learn the relative importance of start/end boundary features and contextual information.

**Table 7:** Performance of different boundary fusion methods.

Fusion Methods	Weibo			OntoNotes5.0		
	*P*(%)	*R*(%)	F1(%)	*P*(%)	*R*(%)	F1(%)
Fixed-Weight Start Position Fusion	65.21	62.47	63.81	76.72	77.54	77.13
Fixed-Weight End Position	66.85	60.74	63.65	76.62	77.94	77.28
Fixed-Weight Joint Position Fusion	68.64	60.00	64.03	78.15	76.55	77.34
Learned-Weight Adaptive Fusion	66.32	62.72	64.47	77.93	77. 64	77.78

The experimental results as shown in Table 7 reveal that models utilizing only start or end position features with fixed weights exhibit significant performance degradation compared to joint fusion methods. Combining both start and end position features consistently improves F1 scores across datasets, indicating the complementary nature of boundary cues. The learned-weights adaptive fusion method outperforms fixed-weight approaches, achieving a 0.44% F1 improvement on both the Weibo and OntoNotes 5.0 datasets. This demonstrates the model’s ability to dynamically prioritize task-relevant boundary information during training. These findings underscore the importance of adaptive feature fusion in leveraging entity boundary information. By allowing the model to automatically learn optimal fusion weights, the proposed approach effectively addresses the variability in entity complexity and contextual ambiguity across different datasets.

## Conclusion

Traditional Chinese NER models face significant challenges, particularly in effectively leveraging entity boundary information. To address this limitation, we propose a novel joint learning framework that unifies boundary prediction and entity classification into a single task.

Compared to traditional sequence labeling methods, our approach offers several key advantages. First, by modeling boundary prediction and entity classification simultaneously, the model achieves more accurate identification of named entity candidates. This integration enhances the model’s understanding of the structural and semantic context surrounding entities, including intra- and inter-entity relationships. Additionally, our method incorporates richer features that capture both the internal structure and external context of named entity candidates, leading to better overall performance.

Our approach has several distinct advantages over traditional sequence labeling approaches. Firstly, instead of dealing with boundary prediction and entity categorization as separate tasks, our method models them simultaneously, leading to a more precise identification of named entity candidates. By integrating these two tasks, the model can gain a more comprehensive understanding of the structural context around named entities, including the semantic relationships within and between entities. Additionally, our approach enables the incorporation of more effective features that capture the inner structure and outer surroundings of named entity candidates. By considering a wider range of features, we enhance the discriminative capability of our model and improve the overall performance.

We conducted experiments on the Weibo and OntoNotes 5.0 datasets, and results show that our model outperforms comparative baselines in Chinese NER. Furthermore, we performed ablation studies to analyze the impact of individual components within our framework. The results confirm that the inclusion of additional entity boundary information contributes significantly to enhancing the model’s final performance.

While the proposed method demonstrates strong performance through multi-task learning of boundary and label information, challenges remain for nested entities, where boundary misjudgments persist. For future work, we aim to integrate external knowledge sources to refine boundary detection and optimize positional encodings. Furthermore, combining the model with complementary tasks like part-of-speech tagging or syntactic parsing is expected to yield synergistic effects, advancing the state of the art in Chinese NER.

## Data Availability

No datasets were generated or analysed during the current study.
